# Creating biobanks in low and middle-income countries to improve knowledge – The PREPARE initiative

**DOI:** 10.1016/j.preghy.2018.05.007

**Published:** 2018-07

**Authors:** Leandro De Oliveira, Marcos Augusto Bastos Dias, Arundhanthi Jeyabalan, Beth Payne, Christopher W. Redman, Laura Magee, Lucilla Poston, Lucy Chappell, Paul Seed, Peter von Dadelszen, James Michael Roberts

**Affiliations:** a Medical School, Botucatu Sao Paulo State University, Obstetrics Department, Brazil; b Fernandes Figueira Institute, Rio de Janeiro, Brazil; c Department of Obstetrics and Gynecology, University of Pittsburgh, Pittsburgh, PA, United States; d Healthy Starts Theme, BC Children’s Hospital Research, Vancouver, BC, Canada; e Department of Anaesthesiology, Pharmacology and Therapeutics, University of British Columbia, Vancouver, BC, Canada; f Nuffield Department of Obstetrics and Gynaecology, University of Oxford, United Kingdom; g St. George’s Hospitals NHS Foundation Trust, University of London, London, United Kingdom; h Division of Women’s Health, Women’s Health Academic Centre, King’s College London and King’s Health Partners, 10th Floor, North Wing, St. Thomas’ Hospital, London, United Kingdom

**Keywords:** Maternal mortality, Pre-eclampsia, Biomarkers, Biobank

## Abstract

The Millennium Development Goal 5, a project signed in 2000, intended to improve maternal health and reduce maternal mortality by 75% by 2015. Despite all efforts, little progress has been achieved in low and middle-income countries (LMIC) and 99% of all maternal deaths related to pre-eclampsia (PE) still occur in these settings. It is important to determine whether women in LMIC, where PE carries a greater risk than in high-income countries (HIC), have unique risk factors. Some variances may alter the risk, severity and pertinent pathophysiology of PE. We posit based upon this, that women from LMIC may have biomarkers specific to this population. Discovering such specific biomarkers and testing the relevance of biomarkers developed in high-income populations could increase the clinical usefulness of these analyses without increasing cost-effective approaches for prediction of PE.

Here we briefly describe our platform to develop the PREPARE – Biobank in tertiary hospitals or basic units for antenatal care from 6 different cities in Brazil.

The PREPARE – Biobank has been developed with two arms. The first arm is a cross-sectional study that will collect clinical information and biosamples from more than 1000 women who developed preterm PE. The second arm is a cohort study of 7000 women. It will collect clinical information and longitudinal biosamples from women at three times during pregnancy, <16 weeks, between 28 and 32 weeks and at delivery or diagnosis of adverse outcomes.

The biobank will be supported and complemented by a Brazilian database using the CoLab COLLECT Database.

## Highlights

• Low and middle-income (LMIC) countries still suffer with pre-eclampsia.
• Risk assessment of pre-eclampsia has evolved in high-income countries.
• Biobanks can provide a unique opportunity to understand preeclampsia in LMIC.
• Databanks can provide a unique opportunity to understand preeclampsia in LMIC.

## 1 Introduction

Data from the World Health Organization (WHO) show that every day, 830 women die from preventable causes related to pregnancy and childbirth, with the vast majority of these deaths (99%) occurring in low and middle-income countries. In addition, among the 133 million babies born alive each year, 2.8 million die in the first week of life. The patterns of these deaths are similar to the patterns for maternal deaths, the majority occurring in developing countries [1].

This sad reality motivated the creation of the Millennium Development Goal 5 (MDG 5), a project signed in 2000 to improve maternal health and reduce maternal mortality by 75% by 2015. Despite all efforts, little progress has been achieved in developing countries. In Brazil for example, the official report on maternal mortality ratio in 1999 was 73. In 2015 this ratio was 58 [2], [3].

Low and middle-income (LMIC) countries still suffer with pre-eclampsia (PE), post-partum hemorrhage and illegal abortions as important causes of maternal deaths. In Latin America, including Brazil, hypertensive disorders, mainly PE, account for 26% of all causes of maternal deaths [4].

It is clear that LMICs benefit from assistance from high resource centres to improve the management of PE. This has begun in Africa and India, with encouraging results [5]. Therefore, our aim is to extend this approach to Brazil and Latin America. We believe that in addition to the usual strategy of implementing approaches that have been successful in high-income countries (HIC), effective management of PE and other adverse pregnancy outcomes in Brazil demands an understanding of the pathophysiology of the disease in Brazil. To enable rational antenatal risk assessment, prophylaxis and treatment, we believe we must consider potentially unique features of the disorder in the local setting. Currently, risk assessment of PE, using demographic and clinical features and biomarkers, has evolved almost exclusively in HIC. This approach has two problems in terms of usefulness for LMIC. Firstly, discoveries developed in high-income populations may not be completely pertinent to other populations. For example while some authors demonstrated 90% sensitivity for prediction of PE using a set of biomarkers in Europe (PAPP-A, PlGF, PP-13 and Doppler of uterine arteries), Oliveira et al. showed only 52% in United States of America [6], [7]. Secondly, platforms developed for HIC maybe too expensive to be useful in LMIC.

Bearing these considerations in mind, it is important to determine whether women in LMIC, where PE carries a greater risk than in HIC, have unique risk factors. Some variances (e.g. diet, smoking status, coital and partnership history) may alter the risk, severity and pertinent pathophysiology of PE compared to HIC. We posit based upon this, that women from LMIC may have biomarkers specific to this population. Discovering such specific biomarkers and testing the relevance of biomarkers developed in high-income populations could increase the clinical usefulness of these analyses without increasing cost-effective approaches for prediction of PE for this population.

The Global Pregnancy Collaboration (CoLab), a group of international researchers from more than 40 countries believes that the creation of a robust biobank linked to a reliable databank is one of the most powerful initiatives, to rapidly and economically, achieve the goal of understanding potential unique features of diseases in the local population. This will thus empower LMICs to achieve the goal of MDG 5. Here we describe our platform to develop the PREPARE – Biobank in Brazil. The PREPARE – (Prematurity REduction by Preeclampsia cARE project) is an audacious project that has been developed either in tertiary hospitals or basic units for antenatal care from 6 different cities in Brazil. It has been supported by an association between Bill & Melinda Gates Foundation and CNPq (Brazilian Government).

The PREPARE – Biobank has been developed with two arms. The first arm is a cross-sectional study that will collect clinical information and biosamples from 1000 women who developed preterm PE. The second arm is a cohort study of 7000 women. It will collect clinical information and longitudinal biosamples from women at three times during pregnancy, <16 weeks, between 28 and 32 weeks and at delivery or diagnosis of adverse outcomes. The population to be included in the PREPARE is comprised mainly of low-income women. The PREPARE – Biobank will provide a unique opportunity to bank biological materials from Brazilian women, different from HIC, for present and future mechanistic studies.

## 2 Purpose of the PREPARE database and biobank

Our purpose is to evaluate women with PE and other adverse pregnancy outcomes using data and materials from this PREPARE database and biobank, searching for unique biological biomarkers and testing biomarkers developed in HIC in the Brazilian population. We believe this will provide insights that may also be pertinent to other LMICs.

## 3 The PREPARE – database

The biobank will be supported and complemented by Brazilian database using the CoLab COLLECT Database (Myatt L, Roberts JM, Redman CWG, Global Pregnancy C. Availability of COLLECT), a database for pregnancy and placental research studies worldwide [8]. This is a very complete database with many defined risk factors for PE and additional information. All data has been recorded and stored in a secure on-line system provided by MedSciNet group. Details including the mother’s own birth-weight and mode of delivery, her length of cohabitation with the father of her child, and many others will be recorded. We expect to collect this high quality demographic, clinical and outcome data for 7000 women from the cohort arm and for more than 1500 women from the cross-sectional arm. A pilot questionnaire about air pollution and PE will be introduced among women recruited with preterm PE in one of the centres involved in the study. The implication of air pollution in the pathophysiology of PE is a recent topic that needs to be addressed.

## 4 The PREPARE – biobank

All women at the cohort arm will provide blood (plasma and serum), urine and DNA samples at ≤16 and 28–32 weeks’ gestation and at the time of delivery or adverse outcomes. This model will allow us to study other adverse outcomes including preterm birth, small for gestational age and others.

Standardized operating procedures (SOPs), approved by the Management Team, was designed to guide recruitment, clinical procedures, and sample collection, processing and storage.

We are providing each antenatal care unit with a tabletop centrifuge and a small −20 °C freezer. Each tertiary centre will have a larger −20 °C freezer and the Biorepository will have seven −80 °C freezers. Serum and plasma will be prepared and aliquoted at the antenatal centre and stored temporarily in small −20 °C freezers with transfer to the associated centre weekly. At the centre, samples will be bar coded, entered into the inventory system and stored at −20 °C. Samples will be transferred monthly on dry ice for long-term storage (−80 °C) at the Study Centre Laboratory. The inventory system information will include the duration of time from draw to processing, entry to −20 °C freezer and time in −20 °C freezer prior to entry to −80 °C freezer.

Facilities for long-term sample storage (Biorepository) will be located at the Study Centre Laboratory, Botucatu São Paulo State University, where seven −80 °C freezers with a nitrogen back-up system purchased for this purpose will be installed in the Laboratory with emergency power and alarms. In the event of freezer malfunction back-up freezers are available at this site for use during freezer repair. We have selected a dispersed system of accrual and temporary storage and centralized long-term storage as the safest and most economical. Maintaining samples at −20 °C is in many settings considered sufficient for long-term storage. Our experience is that long-term (months to years) is more effective at −80 °C but for short-term (weeks) −20 °C is adequate. The availability of the alarm emergency power and back-up capabilities will provide adequate protection. We envision this as the inception of the Biorepository, which we will extend with future funding.

Through CoLab this data and biological materials will be made accessible to the pregnancy research community. This includes LMIC-based investigators who will have access to this rich data set and Biobank for studies that would, at this time, be beyond their practical ability to conduct due to local resource constraints. Through HIC centres in CoLab we will have samples to compare findings in women in Brazil and HIC centres.

We will also work with existing Bill & Melinda Gates Foundation centres and other agencies striving to harmonize data and sample collection in LMICs. The intent is to acquire useful data and samples appropriate for assessment of PE and other pregnancy outcomes. The approach will balance robust data and biomaterials collection with what is achievable in LMICs.

We believe the combined approach of testing the value of established approaches and searching for unique biomarkers and pathophysiological features in the local settings using biobanks and databanks in LMIC will help to achieve the goals of MDG 5. CoLab offers assistance to LMICs for applications to obtain funding for this goal (https://pregnancycolab.tghn.org) and also will provide the COLLECT database for these efforts (https://pregnancycolab.tghn.org/articles/collect-database/).

This work has been approved by the National Central Ethical Committee on research involving Human Subjects (CAAE: 53092916.4.2008.5411) and by each Local Ethical Committee of the participating centres.
